# Supplementary material for: Resolving the full spectrum of human genome variation using Linked-Reads
Source: Genome Res. 2019 Apr;29(4):635–45. doi: 10.1101/gr.234443.118 (PMC6442396; doi:10.1101/gr.234443.118)
Supplement: Supplemental Material [file supp_gr.234443.118_Supplemental_longranger-2.2.zip › longranger-2.2.tar.bz2!/lr2.2-public/lib/go/src/code.google.com/vitess/docs/user-guide/horizontal-sharding-workflow.html]

Vitess / Horizontal Sharding (Tutorial, automated)


Toggle navigation

- Overview
- Guides
- Reference
- Blog
- GitHub

- #### Overview

  - What is Vitess
  - Scaling MySQL with Vitess
  - Key Concepts
- #### Getting Started

  - Run Vitess on Kubernetes
    - Custom Docker Build
  - Run Vitess Locally
- #### User Guide

  - Introduction- Client Libraries- Backing Up Data- Reparenting
        - Schema Management
        - VSchema Guide
        - Schema Swap (Tutorial)
        - Sharding
          - Horizontal Sharding (Tutorial, automated)
          - Horizontal Sharding (Tutorial, manual)
          - Sharding in Kubernetes (Tutorial, automated)
          - Sharding in Kubernetes (Tutorial, manual)
        - Vitess Sequences
        - MySQL Server Protocol
        - Vitess and Replication
        - Update Stream
        - Row Based Replication
        - Topology Service
        - Transport Security Model
        - Launching
          - Scalability Philosophy
          - Production Planning
          - Server Configuration
          - 2PC Guide
          - Troubleshooting
        - Upgrading
- #### Reference Guides

  - Vitess API- vtctl Commands
- #### Other Resources

  - Presentations- Blog- Roadmap
- #### Contributing

  - Contributing to Vitess- GitHub Workflow- Code Reviews
- GitHub

# Horizontal Sharding (Tutorial, automated)

- #### Overview

  - What is Vitess
  - Scaling MySQL with Vitess
  - Key Concepts
- #### Getting Started

  - Run Vitess on Kubernetes
    - Custom Docker Build
  - Run Vitess Locally
- #### User Guide

  - Introduction- Client Libraries- Backing Up Data- Reparenting
        - Schema Management
        - VSchema Guide
        - Schema Swap (Tutorial)
        - Sharding
          - Horizontal Sharding (Tutorial, automated)
          - Horizontal Sharding (Tutorial, manual)
          - Sharding in Kubernetes (Tutorial, automated)
          - Sharding in Kubernetes (Tutorial, manual)
        - Vitess Sequences
        - MySQL Server Protocol
        - Vitess and Replication
        - Update Stream
        - Row Based Replication
        - Topology Service
        - Transport Security Model
        - Launching
          - Scalability Philosophy
          - Production Planning
          - Server Configuration
          - 2PC Guide
          - Troubleshooting
        - Upgrading
- #### Reference Guides

  - Vitess API- vtctl Commands
- #### Other Resources

  - Presentations- Blog- Roadmap
- #### Contributing

  - Contributing to Vitess- GitHub Workflow- Code Reviews

This guide shows you an example about how to apply range-based sharding
process in an existing unsharded Vitess keyspace
using the horizontal resharding workflow. In this example, we will reshard
from 1 shard "0" into 2 shards "-80" and "80-".

## Overview

The horizontal resharding process mainly contains the following steps
(each step is a phase in the workflow):

1. Copy schema from original shards to destination shards.
   (**Phase: CopySchemaShard**)
2. Copy the data with a batch process called *vtworker*
   (**Phase: SplitClone**).
   more details
3. Check filtered replication (**Phase: WaitForFilteredReplication**).
   more details
4. Check copied data integrity using *vtworker* batch process in the mode
   to compare the source and destination data. (**Phase: SplitDiff**)
5. Migrate all the serving rdonly tablets in the original shards.
   (**Phase: MigrateServedTypeRdonly**)
6. Migrate all the serving replica tablets in the original shards.
   (**Phase: MigrateServedTypeReplica**)
7. Migrate all the serving master tablets in the original shards.
   (**Phase: MigrateServedTypeMaster**)
   more details

## Prerequisites

You should complete the Getting Started guide
(please finish all the steps before Try Vitess resharding) and have left
the cluster running. Then, please follow these steps before running
the resharding process:

1. Configure sharding information. By running the command below, we tell
   Vitess to shard the data using the page column through the provided VSchema.

   ```
   vitess/examples/local$ ./lvtctl.sh ApplyVSchema -vschema "$(cat vschema.json)" test_keyspace
   ```
2. Bring up tablets for 2 additional shards: *test\_keyspace/-80* and
   *test\_keyspace/80-* (you can learn more about sharding key range
   here):

   ```
   vitess/examples/local$ ./sharded-vttablet-up.sh
   ```

   Initialize replication by electing the first master for each of the new shards:

   ```
   vitess/examples/local$ ./lvtctl.sh InitShardMaster -force test_keyspace/-80 test-200
   vitess/examples/local$ ./lvtctl.sh InitShardMaster -force test_keyspace/80- test-300
   ```

   After this set up, you should see the shards on Dashboard page of vtctld UI
   (http://localhost:15000). There should be 1 serving shard named "0" and
   2 non-serving shards named "-80" and "80-". Click the shard node, you can
   inspect all its tablets information.
3. Bring up a vtworker process, which can be connected through port 15033.
   (The number of *vtworker* should be the same of original shards,
   we start one vtworker process here since we have only one original shard
   in this example.)

   ```
   vitess/examples/local$ ./vtworker-up.sh
   ```

   You can verify this *vtworker* process set up through http://localhost:15032/Debugging.
   It should be pinged successfully. After you ping the vtworker, please click
   "Reset Job". Otherwise, the vtworker is not ready for executing other tasks.

## Horizontal resharding workflow

### Create the workflow

1. Open the *Workflows* section on the left menu of vtctld UI (http://localhost:15000).
   Click the "+" button in the top right corner to open the "Create
   a new Workflow" dialog.
2. Fill in the "Create a new Workflow" dialogue following the instructions
   below (you can checkout our example here):
   - Select the "Skip Start" checkbox if you don't want to start the workflow
     immediately after creation. If so, you need to click a "Start" button in
     the workflow bar later to run the workflow.
   - Open the "Factory Name" menu and select "Horizontal Resharding". This field
     defines the type of workflow you want to create.
   - Fill in *test\_keyspace* in the "Keyspace" slot.
   - Fill in *localhost:15033* in the "vtworker Addresses" slot.
   - Unselect the "enable\_approvals" checkbox if you don't want to manually
     approve task executions for canarying. (We suggest you to keep the default
     selected choice since this will enable the canary feature)
3. Click "Create" button at the bottom of the dialog. You will see a workflow
   node created in the *Workflows* page if the creation succeeds.
   The workflow has started running now if "Skip Start" is not selected.

Another way to start the workflow is through the vtctlclient command, you can
also visualize the workflow on vtctld UI *Workflows* section after executing
the command:

```
vitess/examples/local$ ./lvtctl.sh WorkflowCreate -skip_start=false horizontal_resharding -keyspace=test_keyspace -vtworkers=localhost:15033 -enable_approvals=true
```

When creating the resharding workflow, the program automatically detect the
source shards and destination shards and create tasks for the resharding
process. After the creation, click the workflow node, you can see a list of
child nodes. Each child node represents a phase in the workflow (each phase
represents a step mentioned in Overview).
Further click a phase node, you can inspect tasks in this phase.
For example, in the "CopySchemaShard" phase, it includes tasks to copy schema
to 2 destination shards, therefore you can see task node "Shard -80" and
"Shard 80-". You should see a page similar to
this.

### Approvals of Tasks Execution (Canary feature)

Once the workflow start to run (click the "Start" button if you selected
"Skip Start" and the workflow hasn't started yet.), you need to approve the
task execution for each phase if "enable\_approvals" is selected. The approvals
include 2 stages. The first stage approves only the first task, which runs as
canarying. The second stage approves the remaining tasks.

The resharding workflow runs through phases sequentially. Once the phase starts,
you can see the approval buttons for all the stages under the phase node (click
the phase node if you didn't see the approval buttons, you should see a page
like this). The
button is enabled when the corresponding task(s) are ready to run. Click the
enabled button to approve task execution, then you can see approved message
on the clicked button. The approval buttons are cleared after the phase has
finished. The next phase will only starts if its previous phase has finished
successfully.

If the workflow is restored from a checkpoint, you will still see the the
approval button with approved message when there are running tasks under this
approval. But you don't need to approve the same tasks again for a restarted
workflow.

### Retry

A "Retry" button will be enabled under the task node if the task failed (click
the task node if your job get stuck but don't see the Retry button). Click this
button if you have fixed the bugs and want to retry the failed task. You can
retry as many times as you want if the task continuelly failed. The workflow
can continue from your failure point once it is fixed.

For example, you might forget to bring up a vtworker process. The task which
requires that vtworker process in SplitClone phase will fail. After you fix
this, click the retry button on the task node and the workflow will continue
to run.

When a task failed, the execution of other tasks under this phase won't be
affected if this phase runs tasks in parallel (applied to phase
"CopySchemaShard", "SplitClone", "WaitForFilteredReplication"). For phases
that runs tasks sequentially, remaining unstarted tasks under this phase will
no long be executed. The phases afterwards will no longer be executed.

### Checkpoint and Recovery

The resharding workflow tracks the status for every task and checkpoint these
status into topology server whenever there is a status update. When a workflow
is stopped and restarted by loading the checkpoint in the topology, it can
continue to run all the unfinished tasks.

## Verify Results and Clean up

After the resharding process, data in the original shard is identically copied
to new shards. Data updates will be visible on the new shards, but not on the
original shard. You should then see in the vtctld UI *Dashboard* page that shard
*0* becomes non-serving and shard *-80* and shard *80-* are serving shards.
Verify this for yourself: inspect the database content using following commands,
then add messages to the guestbook page (you can use script client.sh mentioned
here)
and inspect using same commands:

```
# See what's on shard test_keyspace/0
# (no updates visible since we migrated away from it):
vitess/examples/local$ ./lvtctl.sh ExecuteFetchAsDba test-100 "SELECT * FROM messages"
# See what's on shard test_keyspace/-80:
vitess/examples/local$ ./lvtctl.sh ExecuteFetchAsDba test-200 "SELECT * FROM messages"
# See what's on shard test_keyspace/80-:
vitess/examples/local$ ./lvtctl.sh ExecuteFetchAsDba test-300 "SELECT * FROM messages"
```

You can also checkout the *Topology* browser on vtctl UI. It shows you the
information of the keyrange of shard and their serving status. Each shard
should look like this

shard 0

shard -80

shard 80-

After you verify the result, we can remove the
original shard since all traffic is being served from the new shards:

```
vitess/examples/local$ ./vttablet-down.sh
```

Then we can delete the now-empty shard:

```
vitess/examples/local$ ./lvtctl.sh DeleteShard -recursive test_keyspace/0
```

You should then see in the vtctld UI *Dashboard* page that shard *0* is gone.

## Tear down and clean up

Since you already cleaned up the tablets from the original unsharded example by
running `./vttablet-down.sh`, that step has been replaced with
`./sharded-vttablet-down.sh` to clean up the new sharded tablets.

```
vitess/examples/local$ ./vtworker-down.sh
vitess/examples/local$ ./vtgate-down.sh
vitess/examples/local$ ./sharded-vttablet-down.sh
vitess/examples/local$ ./vtctld-down.sh
vitess/examples/local$ ./zk-down.sh
```

## Reference

You can checkout the old version tutorial here.
It walks you through the resharding process by manually executing commands.

### Details in SplitClone phase

*vtworker* copies data from a paused snapshot. It will pause replication on
one rdonly (offline processing) tablet to serve as a consistent snapshot of
the data. The app can continue without downtime, since live traffic is served
by replica and master tablets, which are unaffected. Other batch jobs will
also be unaffected, since they will be served only by the remaining, un-paused
rdonly tablets.

During the data copying, *vtworker* streams the data from a single source to
multiple destinations, routing each row based on its *keyspace\_id*. It can
automatically figure out which shards to use as the destinations based on the
key range that needs to be covered. In our example, shard 0 covers the entire
range, so it identifies -80 and 80- as the destination shards, since they
combine to cover the same range.

### Details in WaitForFilteredReplication phase

Once the copying from a paused snapshot (phase SplitClone) has finished,
*vtworker* turns on filtered replication,
which allows the destination shards to catch up on updates that have continued
to flow in from the app since the time of the snapshot. After the destination
shards are caught up, they will continue to replicate new updates.

### Details in MigrateServedTypeMaster phase

During the *master* migration, the original shard masters will first stop
accepting updates. Then the process will wait for the new shard masters to
fully catch up on filtered replication before allowing them to begin serving.
After the master traffic is migrated, the filtered replication will be stopped.
Data updates will be visible on the new shards, but not on the original shards.

Contact: vitess@googlegroups.com  **·**  
Announcements  **·**  
© 2017 Vitess powered by Google Inc
